# Acute kidney injury and intra-abdominal hypertension in burn patients in intensive care

**DOI:** 10.5935/0103-507X.20180001

**Published:** 2018

**Authors:** Thalita Bento Talizin, Meiry Sayuri Tsuda, Marcos Toshiyuki Tanita, Ivanil Aparecida Moro Kauss, Josiane Festti, Cláudia Maria Dantas de Maio Carrilho, Cintia Magalhães Carvalho Grion, Lucienne Tibery Queiroz Cardoso

**Affiliations:** 1 Universidade Estadual de Londrina - Londrina (PR), Brazil.

**Keywords:** Intensive care units, Renal insufficiency, Intra-abdominal hypertension, Burn units, Burns, Multiple organ failure

## Abstract

**Objective:**

To evaluate the frequency of intra-abdominal hypertension in major burn patients and its association with the occurrence of acute kidney injury.

**Methods:**

This was a prospective cohort study of a population of burn patients hospitalized in a specialized intensive care unit. A convenience sample was taken of adult patients hospitalized in the period from 1 August 2015 to 31 October 2016. Clinical and burn data were collected, and serial intra-abdominal pressure measurements taken. The significance level used was 5%.

**Results:**

A total of 46 patients were analyzed. Of these, 38 patients developed intra-abdominal hypertension (82.6%). The median increase in intra-abdominal pressure was 15.0mmHg (interquartile range: 12.0 to 19.0). Thirty-two patients (69.9%) developed acute kidney injury. The median time to development of acute kidney injury was 3 days (interquartile range: 1 - 7). The individual analysis of risk factors for acute kidney injury indicated an association with intra-abdominal hypertension (p = 0.041), use of glycopeptides (p = 0.001), use of vasopressors (p = 0.001) and use of mechanical ventilation (p = 0.006). Acute kidney injury was demonstrated to have an association with increased 30-day mortality (log-rank, p = 0.009).

**Conclusion:**

Intra-abdominal hypertension occurred in most patients, predominantly in grades I and II. The identified risk factors for the occurrence of acute kidney injury were intra-abdominal hypertension and use of glycopeptides, vasopressors and mechanical ventilation. Acute kidney injury was associated with increased 30-day mortality.

## INTRODUCTION

The occurrence of intra-abdominal hypertension (IAH) in surgical patients and those with sepsis or trauma has been widely described in the literature.^(1,2)^ Abdominal compartment syndrome (ACS) is a complication resulting from increased intra-abdominal pressure (IAP). High IAP values are not physiologically tolerated and are associated with organ dysfunction, especially of the hemodynamic, respiratory and renal types.^(3)^ Early diagnosis is essential to prevent complications caused by IAH.^(4)^

Given the importance of the topic, the World Society of Abdominal Compartment Syndrome (WSACS) was founded. This organization prepared a document standardizing definitions and normal IAP values to guide clinical practice.^(5)^ Normal IAP values range from 0 to 12 mmHg. Sustained increases in IAP above 12mmHg define IAH. ACS is defined as increases in IAP to above 20mmHg associated with organ dysfunction.

Risk factors associated with ACS development can be classified as primary or secondary. Primary factors include causes that are anatomically located in the pelvis and abdomen. Secondary factors are due to other causes, such as sepsis, acidosis, hypothermia, fluid replacement and systemic inflammatory response. In major burn patients, the presence of thermal injury in the abdomen, capillary leak secondary to systemic inflammatory response and aggressive fluid replacement are factors that contribute to increased IAP.^(6)^

The incidence of IAH in major burn patients is variable in the literature and is associated with the burn area; it is higher in patients with burns covering more than 20% of the body surface area.^(7)^ The use of mechanical ventilation is also associated with an increased incidence of IAH and to a worse prognosis in untreated cases.^(8)^

In major burn patients, IAH generally occurs in the first 48 hours of the initial resuscitation period. ACS occurs after the acute phase and is associated with episodes of infectious complications.^(9)^ The kidneys are very vulnerable organs during the initial treatment of major burns, whether due to the occurrence of IAH, surgical intervention or the presence of nephrotoxic agents. Acute kidney injury (AKI) may result from the reduction in renal blood flow in cases of IAH; in this scenario, urine flow cannot be used as a fluid replacement guide, leading to the loss of an important major burn monitoring parameter.

The objective of this study was to evaluate the frequency of IAH in major burn patients and its association with the occurrence of acute kidney injury.

## METHODS

This study was approved by the Research Ethics Committee of the *Hospital Universitário Regional do Norte do Paraná - Universidade Estadual de Londrina* under CEP 041/2013, CAAE 13327013.8.0000.5231. All study participants agreed with the research and signed an informed consent form.

This was a prospective cohort study. The study population consisted of patients hospitalized in specialized intensive care unit (ICU) beds in the Burn Treatment Center of a university hospital.

A convenience sample was taken of adult burn patients consecutively admitted at the study site. All those admitted between August 2015 and October 2016 were included. Patients under 18 years of age, those with a burned body surface area of less than 20%, those diagnosed with burn-associated trauma and those who did not consent to participate were excluded. Data pertaining to patients included in the study were collected during their ICU stay, and the date of and outcome at hospital discharge were recorded.

Data collection included clinical, laboratory and demographic data, primary and secondary diagnoses and data on burn type and extent. Data concerning the nephrotoxic drugs used during the ICU stay were also collected. Patient severity was evaluated using the Abbreviated Burn Severity Index (ABSI) score.^(10)^

The burned body surface was calculated based on the Lund and Browder chart^(11)^ by a plastic surgery specialist at hospital admission. Accumulated fluid balance was defined as the result of the sum of the daily recording of infused fluids and fluids eliminated by the patient within the first 48 hours. IAH was defined, according to WSACS criteria, as a sustained or repeated IAP increase of ≥ 12mmHg. IAH was classified into grades, according to IAP values, and scaled as grade I (12 - 15mmHg), grade II (16 - 20mmHg), grade III (21 - 25mmHg) and grade IV (> 25mmHg). ACS was defined as a sustained IAP value of > 20mmHg associated with new organ failure or dysfunction.^(5)^ AKI was defined as increased baseline creatinine equal to or greater than 0.3mg/dL within 48 hours or greater than or equal to 1.5 times within a 7-day interval.^(12)^

The initial IAP measurement was taken within 3 hours of admission. If the measurement produced a value within normal limits, the IAP was recorded daily, in the morning, always at the same time, for 7 days or until urinary catheter withdrawal. When the mean was > 12mmHg, it was recorded every 6 hours while it remained high.

The IAP was ascertained from the intravesical pressure. The IAP measurement technique was applied using the AbViser^®^ measurement system (ConvaTec),^(13)^ which allows continuous monitoring of IAP, reducing the time for each recording, reducing the margin of error for each measurement and reducing the risk of contamination of the urinary catheter. The system is positioned aseptically between the urinary catheter and the collection system. The patient remains in the supine position without abdominal contraction. The probe is zeroed and positioned on the iliac crest to the level of the mid-axillary line. Sterile saline is drawn through a sterile syringe, secured and connected to a bottle that is in a closed system with the AbViser^®^ Autovalve^®^ device, and 20mL is injected into the bladder, automatically closing the valve to take the IAP reading. The IAP reading taken is shown on a multiparameter monitor at the end of expiration. IAP reading lasts 1 to 3 minutes, and after this period, the valve system opens automatically, and the reading is zeroed. After each reading, it was confirmed that the urine was draining normally.

The results of continuous variables were described using medians and interquartile ranges (ITQ). Categorical data were expressed as frequencies and presented in tables. Categorical variables were analyzed using the chi-squared test. Correlations were ascertained using Pearson's test to evaluate the degree of dependence between variables. Univariate analysis was performed to identify factors associated with an outcome considered to be AKI. Mortality was described using frequencies. A Kaplan-Meier survival curve analysis was performed, and differences between groups were evaluated using the log-rank test. The significance level used was 5%, and the analyses were performed using the MedCalc program for Windows, version 9.3.2.0 (MedCalc Software, Mariakerke, Belgium).

## RESULTS

**Figure 1:**
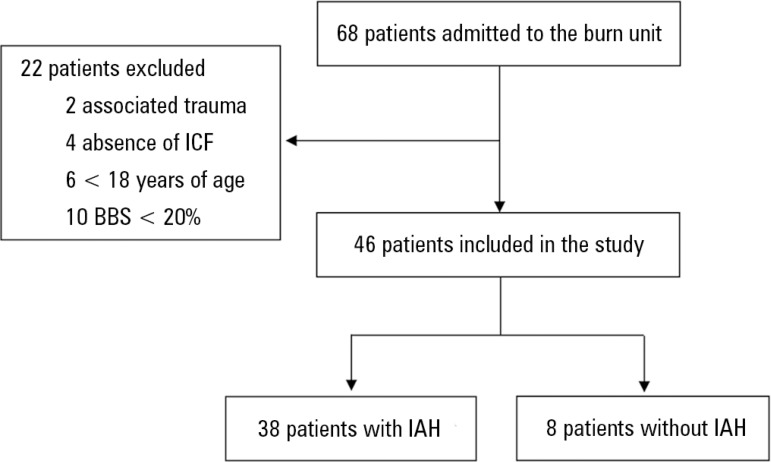
Selection of burn patients admitted to a specialized intensive care unit at a university hospital, 2015-2016. ICF - informed consent form; BBS - burned body surface; IAH - intra-abdominal hypertension.

A total of 68 patients were admitted during the study period. Twenty-two patients were excluded from the study, leaving 46 patients for analysis (Figure 1). Of these, 33 (71.1%) were male; the median age was 40.5 years (ITQ: 28.0 to 53.0). Burns occurred more frequently in domestic accident situations (43.5%), and the median burned body surface area was 30.5% (ITQ: 20.5 to 47.0), as shown in table 1.

**Table 1:** Characterization of hospitalized burn patients admitted to a specialized intensive care unit

Characteristics	N (%)
Age group (years)	
18 - 30	13 (28.3)
31 - 50	21 (45.6)
51 - 70	9 (19.6)
≥ 71	3 (6.5)
Gender	
Female	13 (28.3)
Male	33 (71.1)
Burn agent	
Alcohol	35 (76.1)
Others	11 (23.9)
Burn etiology	
Thermal	39 (84.8)
Electrical	3 (6.5)
Scalding	2 (4.3)
Chemical	2 (4.3)
Context of burning	
Domestic accident	20 (43.5)
Workplace accident	12 (26.1)
Attempted suicide	6 (13.0)
Attempted homicide	5 (10.9)
Fire	3 (6.5)
Presence of Acute Kidney Injury	
Yes	32 (69.9)
No	14 (30.4)
Presence of intra-abdominal hypertension	
Yes	38 (82.6)
No	8 (17.4)
Degree of intra-abdominal hypertension	
No intra-abdominal hypertension	8 (17.4)
Grade I	17 (37.0)
Grade II	12 (26.1)
Grade III	8 (17.4)
Grade IV	1 (2.2)
Presence of abdominal compartment syndrome	
Yes	11 (23.9)
No	35 (76.1)
Use of glycopeptides	
Yes	28 (60.9)
No	18 (39.1)
Use of polymyxin	
Yes	11 (23.9)
No	35 (76.1)
Use of vasopressors	
Yes	33 (71.7)
No	13 (28.3)
Use of mechanical ventilation	
Yes	39 (84.8)
No	7 (15.2)
Outcome at hospital discharge	
Survival	21 (45.7)
Did not survive	25 (54.3)

The median accumulated fluid balance 48 hours after hospitalization was 5,233.0 ml (ITQ: 3,562.0 to 8,224.0). In terms of hospital outcome, 21 (45.7%) patients survived. The median length of stay in the ICU was 15 days (ITQ: 6.0 to 26.0), and the median hospital stay was 20 days (ITQ: 11.0 to 32.0).

A total of 38 (82.6%) patients developed IAH, with grade I being predominant, with 17 cases (37.0%), followed by grade II, with 12 cases (26.1%). ACS developed in 11 patients (23.9%). Comparison of patient group characteristics revealed that the patients who developed IAH had a higher mean age, more severe burns according to the ABSI, developed AKI more frequently and needed to use glycopeptides, vasopressors and mechanical ventilation. The presence of IAH was also associated with a higher mortality rate (Table 2).

**Table 2:** Comparison of clinical characteristics and outcome of burn patients with and without intra-abdominal hypertension admitted to a specialized intensive care unit

Characteristics	With IAH (n=38)	Without IAH (n=8)	p value
Age, years	44 (31 - 54)	30 (23 - 37.5)	0.026
Female	9 (23.7)	4 (50)	0.196
ABSI	8 (7 - 9)	6.5 (5.5 - 7)	0.046
BBS (%)	31 (21 - 47.5)	24.25 (19.75 - 42.25)	0.505
48-hour FB (L)	5,370 (3,857.25 - 8,828.25)	3,894 (2,411 - 5,946)	0.091
Presence of acute kidney injury	29 (76.3)	3 (37.5)	0.044
Use of glycopeptides	26 (68.4)	2 (25)	0.042
Use of polymyxin	9 (23.7)	2 (25)	1.000
Use of vasopressors	30 (78.9)	3 (37.5)	0.031
Use of mechanical ventilation	36 (94.7)	3 (37.5)	0.001
Length of hospitalization	19.5 (7 - 32)	23 (17 - 31.5)	0.310
In-hospital mortality	24 (63.2)	1 (12.5)	0.016

IAH - intra-abdominal hypertension; ABSI - Abbreviated Burn Severity Index; BBS - burned body surface; 48-hour FB - accumulated 48-hour fluid balance. Results are expressed as N (%) or as medians and interquartile ranges.

The peak IAP value showed weak positive correlations with the accumulated fluid balance in the first 48 hours (r = 0.29; p = 0.047) and the worst serum creatinine value during the ICU stay (r = 0.47; p = 0.001).

Of the patients studied, 32 (69.9%) developed AKI during the study period. The median peak serum creatinine value of patients during their ICU stay was 1.33mg/dL (ITQ: 1.0 - 2.39). The median time to developing AKI was 3 days (ITQ: 1 - 7). Univariate analysis of AKI risk factors indicated associations with IAH (p = 0.041), use of glycopeptides (p = 0.001), use of vasopressors (p = 0.001) and use of mechanical ventilation (p = 0.006) (Table 3).

**Table 3:** Univariate analysis of acute kidney injury risk factors in burn patients admitted to a specialized intensive care unit

Variables	Odds ratio	95% CI	p value
Age	1.07	0.96 - 1.20	0.189
Female	0.37	0.09 - 1.44	0.152
ABSI	0.63	0.22 - 1.82	0.400
BBS	1.03	0.90 - 1.17	0.646
Accumulated 48-hour FB	1.00	0.99 - 1.00	0.258
Use of glycopeptides	13.09	2.84 - 60.30	0.001
Use of polymyxin	5.90	0.67 - 51.59	0.108
Use of vasopressors	12.60	2.77 - 57.27	0.001
Use of mechanical ventilation	23.25	2.43 - 221.74	0.006
Intra-abdominal hypertension	5.37	1.06 - 27.00	0.041

95% CI - 95% confidence interval; ABSI - Abbreviated Burn Severity Index; BBS - burned body surface; FB - fluid balance.

**Figure 2:**
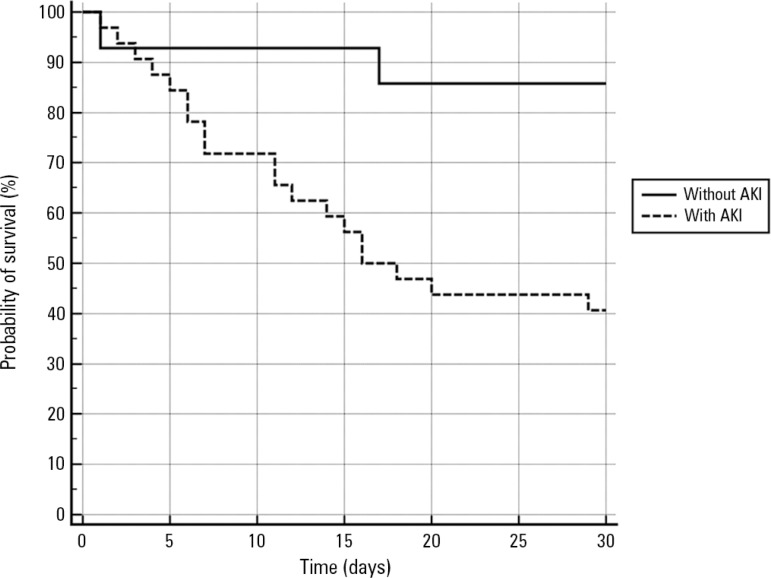
Comparison of 30-day survival between patients with and without acute kidney injury in burn patients admitted to a specialized intensive care unit at a university hospital, 2015-2016. AKI - acute kidney injury. Log-rank, p = 0.009.

The survival analysis (Figure 2) revealed an association between AKI and higher 30-day mortality (log-rank, p = 0.009).

## DISCUSSION

The present study demonstrates the high frequency of IAH in major burn patients and its association with the occurrence of AKI. These results highlight the importance of controlling IAP and preventing AKI in burn patients. Furthermore, they suggest that prevention of AKI occurrence in these patients should lead to an improved mortality rate, as there is an association between AKI and worse prognosis.

The clinical characteristics of the patients in this study are similar to those found in data from other countries. In the United States, the majority of burn patients treated between 2006 and 2015 were male and predominantly between 20 and 59 years old. Domestic occurrences were most prevalent, comprising 73% of cases. The predominant etiology was thermal and by scalding, comprising 75% of patients. Mortality was lower in this US study and increased according to age and greater percent body area burned.^(14)^

In Brazil, males are the most affected, and alcohol is the main agent of burns in adults, predominantly involving domestic accidents.^(15)^ This finding suggests that a high percentage of burns are preventable, with valid prevention measures leading to the avoidance of injury and all of its direct complications and those resulting from treatment. Therefore, it would be appropriate to develop public policies for the prevention of burn accidents and to conduct studies to map the epidemiology of burn accidents in the various regions of the country.

The measurement of IAP has been increasingly performed in the ICU due to the knowledge that has been gained in regard to organ dysfunction resulting from changes in its value.^(16)^ There are variations in techniques used to measure IAP depending on the materials used, but all forms studied involve maintaining the patient in the supine position, without abdominal contraction and with measurement at the end of expiration. The nursing professional who is responsible for setting up the equipment and taking the measurements requires theoretical and practical training to perform this procedure properly. There is a lack of knowledge among health professionals in regard to IAP measurement methodology^(17)^ and a lack knowledge about IAH and its clinical implications.^(18)^ There is still no consensus on a standardized methodology to measure IAP, but there are strong recommendations on the importance of this measurement and its clinical significance for hospitalized patients.^(19)^

The risk factors found for AKI are related to the pathophysiology of kidney injury. The use of nephrotoxic drugs, such as glycopeptides, is associated with direct kidney injury and the consequent dysfunction of this organ, especially if the patient is in the ICU, where serum levels of the drug are above normal and drug treatment is prolonged.^(20,21)^ Changing organic perfusion in the case of circulatory instability, as evidenced in the literature,^(2)^ is a risk factor for kidney injury. The IAH patient also presents hemodynamic changes with impaired renal perfusion.^(9,17)^ The use of mechanical ventilation with consequent changes in intrathoracic pressure is also associated with the presence of IAH. This risk factor is proportional to the severity of respiratory symptoms and the mechanical ventilation requirement.^(22)^

An association between AKI and higher 30-day mortality in intensive care patients has been found.^(23)^ IAH is a complication associated with organ dysfunction, especially AKI, which is a major marker of morbidity and worsening prognosis in ICU patients. Several factors are associated with the development of AKI in-hospital, especially in critically ill patients. Constant IAP measurement can provide proactive information, alerting the team about the imminence of IAH and thus preventing increased morbidity in hospitalized patients.

This study has some limitations, such as the small number of patients and the fact that it is a single-center study. The effects of predictor variables for the outcomes studied may have been underestimated and must be interpreted with caution. The strength of this study is the fact that it is one of the few reports on IAP monitoring in burn patients in Latin America and offers unprecedented local data on the occurrence of IAH and AKI in these patients.

## CONCLUSION

Intra-abdominal hypertension occurred in most patients, predominantly grades I and II. The identified risk factors for the occurrence of acute kidney injury were intra-abdominal hypertension and the use of glycopeptides, vasopressors and mechanical ventilation. Acute kidney injury was associated with higher 30-day mortality in the studied patients.
